# Land use and land cover (LULC) performance modeling using machine learning algorithms: a case study of the city of Melbourne, Australia

**DOI:** 10.1038/s41598-023-40564-0

**Published:** 2023-08-19

**Authors:** Jagannath Aryal, Chiranjibi Sitaula, Alejandro C. Frery

**Affiliations:** 1 Earth Observation and AI Research Group, Department of Infrastructure Engineering, The University of Melbourne, Melbourne, 3053 Australia; 2 School of Mathematics and Statistics, Victoria University of Wellington, Wellington, 6012 New Zealand

**Keywords:** Environmental impact, Computational science

## Abstract

Accurate spatial information on Land use and land cover (LULC) plays a crucial role in city planning. A widely used method of obtaining accurate LULC maps is a classification of the categories, which is one of the challenging problems. Attempts have been made considering spectral (*Sp*), statistical (*St*), and index-based (*Ind*) features in developing LULC maps for city planning. However, no work has been reported to automate LULC performance modeling for their robustness with machine learning (ML) algorithms. In this paper, we design seven schemes and automate the LULC performance modeling with six ML algorithms-Random Forest, Support Vector Machine with Linear kernel, Support Vector Machine with Radial basis function kernel, Artificial Neural Network, Naïve Bayes, and Generalised Linear Model for the city of Melbourne, Australia on Sentinel-2A images. Experimental results show that the Random Forest outperforms remaining ML algorithms in the classification accuracy (0.99) on all schemes. The robustness and statistical analysis of the ML algorithms (for example, Random Forest imparts over 0.99 F1-score for all five categories and *p* value $\le$ 0.05 from Wilcoxon ranked test over accuracy measures) against varying training splits demonstrate the effectiveness of the proposed schemes. Thus, providing a robust measure of LULC maps in city planning.

## Introduction

Land Use and Land Cover (LULC) information derived from remote sensing images is widely used for the aggregation of spatial information, e.g., for the preparation of maps. Firstly, due to the public availability of remote sensing images. Secondly, the analytical capabilities with add-on machine learning algorithms in the public domain. The maps serve as the foundational spatial layer in smart city planning, environmental management, sustainable development, and resilient infrastructure design. The extraction of LULC information is governed by their similarities and dissimilarities in spectral, textural, geometric, and contextual properties stacked in the images. However, the dissection of such properties in an objective and automated manner assists in extracting precise LULC information. Due to the wide application of LULC information and derived maps in smart city planning, their quality needs to be assessed. In LULC classification, deep learning (DL)-based methods in recent studies^1–3^ have resulted in significant performance improvements. Nevertheless, these methods are not only ineffective/inefficient on limited datasets but also have limited interpretability and explainability. Therefore, the use of non-deep learning-based methods^4,5^, which leverage handcrafted geospatial information such as spectral and statistical-based features for the classification, has been preferred to deal with these limitations.

The quality of LULC maps derived from remote sensing images can be characterised using spectral, statistical, and indices-based features. These features and their various combinations can further be assessed in an automated way using state-of-the-art machine learning (ML) algorithms, thus providing quality assurance of the derived products. The performance of employed ML algorithms provides the reliability and assurance of the LULC maps for their use in smart city planning, among many other applications. Thus, derived reliable maps can further be used for balanced sustainable city development and mapping of the coverage of urban green space^6,7^. Despite the usefulness of LULC maps in sustainable development, accurate and precise delineation and detection of LULC in urban settings from remotely sensed images is challenging due to dense physical arrangements and composition of the features, proximity to infrastructure, and complex distribution of assets in multiple hierarchies.

Therefore, precise characterisation of the spatial, spectral, and statistical properties of images using understandable and applicable algorithms and deriving accurate LULC is an open challenge for the spatial information and planning research community. However, with the advancement of ML algorithms, there are potential capabilities that need convergence for a replicable, interpretable, and explainable approach. To contribute to overcoming the existing problems and tackling the challenges, we focus in this study on three key features namely: statistical pattern of reflectance under different bands, spectral information, and leveraging the spectral indices (which are calculated using different spectral bands). Real-world features in images primarily inherit three complementary sources of information (statistical, spectral, and indices-based) which characterise the objects in different dimensions. Characterising this information by dissecting their interrelationships and revealing the redundancy associated with them improves the separability and delineation of the frontiers of LULC classes. Thus, guiding and helping smart city planning practitioners and researchers.

This article’s main contributions are four-fold:

1. We design seven different schemes of characterisation of LULC and classify them with five traditional ML algorithms and one deep learning-based algorithm with a total of six algorithms. Traditional ML algorithms are Random Forest (RF), Support Vector Machine (SVM) with Linear kernel, SVM with Radial Basis Function (RBF) kernel, Naïve Bayes (NB), and Generalized Linear Model (GLM). The DL-based algorithm is Artificial Neural Network (ANN). For the evaluation purpose, we prepare a land cover ground truth (actual) dataset of the City of Melbourne, Victoria, Australia.
2. We perform the category-wise analysis of the best-performing scheme (*Sp*) + (*St*) + (*Ind*) used in our work. Here, we also study the importance of variables in the feature combination along with the model interpretability for generalisability at the category level.
3. We carry out the detailed robustness analysis of ML algorithms using varying training splits at the category level and overall performance level.
4. We conduct pairwise comparisons between ML algorithms on performance measures using statistical analysis.

This paper is organised into five sections. Section “Related works” briefly explains recent feature extraction methods that have been used in LULC classification. Section “Materials and methods” presents the study area in detail, the dataset, and the proposed method. Section “Results and discussion” analyses and discusses LULC classification and ML algorithms’ performance. Finally, section “Conclusion and future works” draws conclusions and presents recommendations.

## Related works

We divide the related works into two broad sections: deep learning-based, and non-deep learning-based feature extraction.

### Deep learning-based feature extraction

Deep learning (DL) is one of the popular ML algorithms, which provides highly discriminating semantic information through its intermediate layers. The applications of DL models span from scene image representation^8,9^, text mining^10^, and biomedical signal analysis^11^, to various remote sensing areas^1,3,12^. For example, Scot et al.^1^ and Zhang et al.^12^ proposed a DL model to classify LULC on very high-resolution (VHR) images. In their method, they used deep features based on the pixel of the images, which significantly improved the performance. Their results showed that it outperforms the traditional ML algorithms (e.g., Support Vector Machine) and some DL-based methods (e.g., U-Net, Convolutional Neural Networks, etc.). The adoption of DL-based methods kept on increasing in the following years after 2018, which further realised the importance of the patch and object of the image. So, Carranza et al.^2^ proposed to use the Convolutional Neural Network (CNN) for the classification of LULC on hyperspectral images and compared with the traditional ML algorithms such as SVM, RF, and k-NN. Furthermore, researchers focused on the different aspects of DL models ranging from sequence extraction to transfer learning based on the pre-trained models to achieve highly discriminating information during classification. For example, Rajendran et al.^13^ employed the long short-term memory (LSTM) model with particle swarm optimization (PSO) for the classification of LULC on Sat 4, Sat 6, and Eurostat datasets. Their model leveraged the texture feature to train the model, which suggested that the combination of LSTM and PSO outperforms LSTM standalone. Furthermore, Hong et al.^14^ proposed a multi-modal feature fusion approach on their newly designed benchmark dataset for LULC classification. Their feature extraction was based on the pixel of the benchmark image datasets, calculating the shared and specific features representing each input image. Following the efficacy of attention information, , Martini et al.^15^ proposed a self-attention-based DL model for LULC classification on Sentinel-2 satellite images. Furthermore, Dewangkoro et al.^16^ developed CNN on EuroSat image datasets for the LULC classification, where they used deep features extracted from such models during classification. In addition, they adopted a squeeze excitation block to extract the salient regions present in the image for better classification.

Recently, Trujillo et al.^17^ employed a DL model for LULC classification using pixel-based features on Sentinel-2 image datasets. Their results suggested that the proposed method provides good performance, outperforming seven traditional ML algorithms (Nearest Neighbors, RF, Multi-layer Perceptron, Adaptive Boosting, NB, Quadratic Discriminant Analysis, and Decision Tree (DT)). Furthermore, Yuan et al.^18^ developed a novel dataset for the LULC classification, where they utilised a DL method for the classification. As a part of feature extraction, they utilised deep features during classification. Wang et al.^19^ proposed a novel DL model, called MLFC-net for LULC classification. Furthermore, Li et al.^20^ developed a multi-scale CNN for LULC classification. Recently, researchers employed deep learning-based zero-shot learning to work with a limited dataset^21,22^ for the LULC classification using VHR images. Similarly, Zhao et al.^23^ devised a transformer and CNN-based approach for the classification of VHR images. Their model leveraged deep features and an attention module to extract meaningful information, which resulted in significantly higher classification performance.

### Non-deep learning-based feature extraction

There are broadly four different types of features under non-deep learning-based feature extraction relevant to LULC classification, according to their source: spectral features, spatial features, thermal features, and temporal features. Spectral features impart the information for each corresponding band in the form of reflectance values. Among others, Wang et al.^4^ and Xu et al.^5^ used such features for different types of images such as Landsat-8 images to represent and classify land covers. Similarly, Bechtel et al.^24^ used spatial features with topographic and structural information. Furthermore, Chen et al.^25^ relied on temporal features based on time stamps to mitigate the misclassification of vegetation types during classification. Further, Pal et al.^26^ conducted a study to observe the LULC’s relationship with the surface temperature using a regression approach in the English Bazar city centre, West Bengal, India. They used different kinds of features such as spectral features, NDWI, and NDVI. Given the importance of thermal radiance, Rodriguez et al.^27^ adopted thermal and multi-spectral features to improve the land-cover classification performance. Yang et al.^28^ employed the DT algorithm for LULC classification on Landsat-8 data using the pixel information of the image. Their results unveiled that their proposed approach outperforms the original decision with the traditional pixel without removing the pixel influence. Zhu et al.^29^ proposed to use of different traditional ML algorithms and DL-based methods for landslide susceptibility detection. Alshari et al.^30^ conducted a survey study about LULC classification focusing on various classes such as water, metropolitan, and woodland from 1950 till now. In their survey study, they highlighted the use of spectral features, object-based features, and hybrid features for the classification by pixel-based approach, object-based approach, and hybrid approach. Zhang et al.^31^ proposed to use feature normalisation before the classification of satellite images into different categories. In their work, texture features using grey-level co-occurrence matrices were extracted from each image and classified them using an SVM and Artificial Neural Network (ANN). Bui et al.^32^ employed the RF and DT algorithms for the classification of LULC classification on Landsat data using pixel-based and object-based features. Their results underlined that the proposed approach could provide a highly accurate output map with efficiency gain in reproducibility time and cost. Furthermore, Hao et al.^33^ developed a LULC cover change detection model in Nyingchi County on China’s Tibetan Plateau on Landsat data using the curved surface area information.

**Figure 1:**
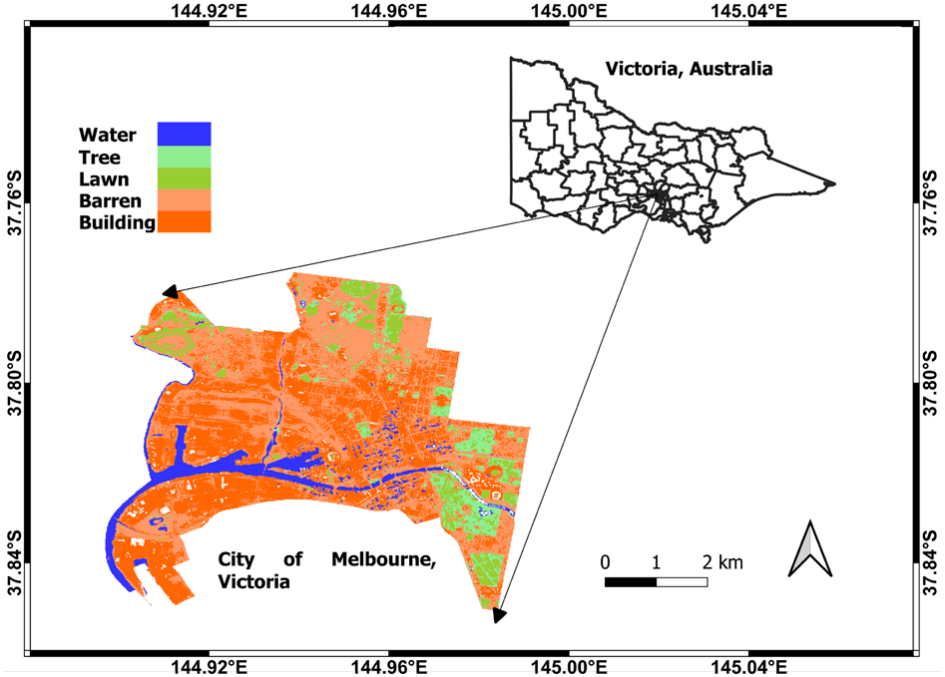
Study area is the City of Melbourne, Victoria, Australia with the mapped LULC of five categories: Water, Tree, Lawn, Barren, and Building, which are drawn using QGIS software^34^ (https://qgis.org/en/site/, version: 3.24.2).

Furthermore, Aryal et al.^35^ proposed to use simple pixel-based information using NDVI threshold for the LULC classification of different LGAs of Victoria, Australia on Sentinel-2A images. Their method suggested that a simple NDVI-based approach has a higher interpretability in developing city greenness projects. Furthermore, Zhao et al.^36^ classified the grasslands in Zambian territory using the optimal combination of spectral bands, feature indices, and topographic features that are selected from a feature importance pipeline. In their work, they used the RF algorithm to classify the grasslands and Google Earth Engine to perform the NDVI time series curve analysis. Hanson et al.^37^ utilised different base ML algorithms to design the ensemble model for the LULC classification on Sentinel-2 data, utilising different spectral bands, spectral indices, and topographic information for each pixel present in the image for the feature extraction. Tang et al.^38^ proposed the two-level classification approach for the land cover changes in cultural heritages on VHR images. They utilised the pixel-level information representing each image and classified it using the RF algorithm. Moreover, He et al.^39^ carried out a study on LULC mapping with the help of pixels and phenological metrics (e.g., maximum NDVI, minimum NDVI, etc.) on MODIS and Google Earth imagery datasets. In their work, they also used additional feature extraction techniques such as texture features for the further improvement of the classification accuracy.

Despite having an excellent performance for the LULC classification, existing methods discussed above exhibit limited explainability/interpretability, resulting in difficulty for the implementation in real applications. Therefore, the convergence of DL and non-DL-based methods would lead to a replicable, interpretable, and explainable approach. The approach we proposed in this paper is novel by utilising three inherent properties of any landscape features namely, spatial, statistical, and index-based (a combination of spatial and statistical properties). Among such features, Xu et al.^5^ flagged that spectral features are the most important for the LULC classification. Because of this result, we further explore such features in this study. For this, along with the spectral features, we leverage spectral indices and statistical features inherent in spectral features to extract the hidden relationships among reflectance values.

**Table 1:** Sample size of ground reference data for each category and their split on train (70 %) and test (30 %) fold.

Category	Building (B)	Barren (Ba)	Lawn (L)	Tree (T)	Water (W)
Train/Test	1672/716	1460/625	1108/474	1232/528	1227/525
Total	2388	2085	1582	1760	1752

**Figure 2:**
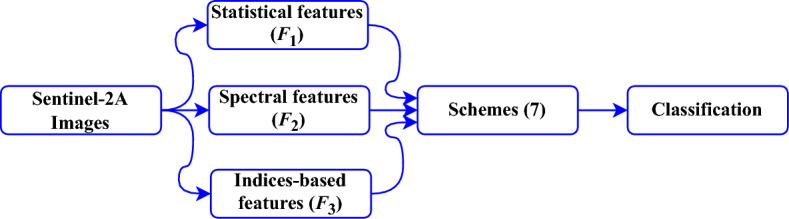
Proposed workflow of our work. We have seven different schemes based on $F_1$, $F_2$, and $F_3$.

## Materials and methods

### Study area

Our study area is the City of Melbourne, which is one of the LGAs (Local Government Areas) of Victoria, Australia. Its geo-location is ${37.50}^\circ {\hbox {S}}$ and ${144.56}^\circ {\hbox {E}}$, and it occupies around ${37}\,{\hbox {km}}^2$. This area’s main land cover types include water, lawn, building, barren, and trees. Further detailed information about the City of Melbourne, Victoria is presented in Fig. 1.

### Satellite images: sentinel-2A

We utilise Sentinel-2A images received from Copernicus Open Access Hub repository^40^. The images were radiometrically, atmospherically, and geometrically corrected to achieve the bottom of atmosphere (BOA) reflectance product, and projected in the Universal Transverse Mercator (UTM)/WGS84 projection system^41,42^. The BOA reflectance image contains 11 bands excluding bands 8 and 10; these two bands were eliminated owing to their lesser importance^43,44^. For our experimental design, we collect 9.567 ground truth (the number of pixels in each class and category) instances using Google Earth Pro software and overlaid them over the Sentinel-2A BOA image from the study area to achieve the corresponding spectral features.

### Implementation

We use R programming language^45^ for the implementation of our whole research work. Further, we exploit R-based packages such as caret^46^ and raster^47^ for ML algorithms and spatial data processing, respectively. We also employ Quantum Geographic Information System (QGIS)^34^ for the visualisation of LULC maps.

For training the ML algorithms, we prepare 10 different random train/test splits of ground truth data (actual), each with the ratio of $70\%/30\%$ (Table 1), and report the averaged performance over such splits (also called Monte Carlo cross-validation) for the evaluation.

**Table 2:** Detailed information about the seven schemes used in this work.

Scheme	Sp	St	Ind	Sp+St	Sp + Ind	St + Ind	Sp + St + Ind
Feature size	11-D	4-D	6-D	15-D	17-D	10-D	21-D
Feature set	[$F_1$]	[$F_2$]	[$F_3$]	[$F_1$,$F_2$]	[$F_1$,$F_3$]	[$F_2$,$F_3$]	[$F_1$,$F_2$,$F_3$]

Sp, St, and Ind are the abbreviated forms of Spatial features, Statistical features, and Indices-based features, respectively.

**Table 3:** Comparative results of six different ML algorithms using classification accuracy ± standard deviation.

	A1	A2		A3	A4	A5	Avg.
Scheme		Lin.	RBF				
Sp	**0.99 ± 0.00**	0.96 ± 0.01	0.97 ± 0.00	0.81 ± 0.07	0.92 ± 0.01	0.95 ± 0.02	0.93
St	**0.99 ± 0.00**	0.92 ± 0.01	0.96 ± 0.01	0.75 ± 0.10	0.93 ± 0.01	0.92 ± 0.01	0.91
Ind	**0.99 ± 0.00**	0.94 ± 0.01	0.95 ± 0.01	**0.92 ± 0.02**	0.93 ± 0.01	0.94 ± 0.00	0.94
Sp+St	**0.99 ± 0.00**	**0.97 ± 0.00**	0.97 ± 0.01	0.83 ± 0.07	0.93 ± 0.01	0.95 ± 0.00	0.94
Sp+Ind	**0.99 ± 0.00**	0.96 ± 0.01	**0.98 ± 0.00**	0.77 ± 0.01	**0.95 ± 0.01**	0.96 ± 0.00	0.93
St+Ind	**0.99 ± 0.00**	0.95 ± 0.00	0.97 ± 0.00	0.77 ± 0.10	0.94 ± 0.01	**0.97 ± 0.00**	0.93
Sp+St+Ind	**0.99 ± 0.00**	**0.97 ± 0.00**	**0.98 ± 0.00**	0.87 ± 0.10	**0.95 ± 0.01**	0.96 ± 0.00	**0.95**

Note that A1, A2, A3, A4, and A5 denote RF, SVM with Linear kernel, SVM with RBF kernel, ANN, NB, and GLM algorithms, respectively. Significant values are in bold.

### Proposed workflow

The proposed workflow (Fig. 2) is divided into five steps: Statistical feature extraction; Spectral feature extraction; Indices-based feature extraction; Schemes; and Classification. The following sections detail these steps.

Let $f:S\rightarrow \mathbb {R}^p$ be an image defined on the grid *S* of dimension $m\times n$, i.e., $S=\{1,2,\dots ,m\}\times \{1,2,\dots ,n\}$, where *p* is the number of bands. Further, let us assume that the image $f:S\rightarrow \mathbb {R}^p$ inherits statistical *St*, spectral *Sp*, and indices *Ind* features.

#### Statistical feature extraction

We extract the basic statistical information such as mean, maximum, minimum, and standard deviation based on spectral reflectance values. Based on such information, we capture the statistical pattern of reflectance values across all bands (Eq. 1). It provides 4-D information on features in the form of statistical feature set $F_1$.

1 $$\begin{aligned} F_1= \{a, b, c, d\}, \end{aligned}$$

where *a*, *b*, *c*, and *d* denote the mean, max, min, and standard deviation of spectral features.

#### Spectral feature extraction

Here, we extract the reflectance values from all the spectral bands for the corresponding location. Each reflectance value under the corresponding spectral band provides the respective reflectance ability (Eq. 2). It provides 11-D information on features in the form of spectral feature set $F_2$.

2 $$\begin{aligned} F_2= \{f_1, f_2, \dots , f_{11}\}, \end{aligned}$$

where $\{f_i\}_{i=1}^{11}$ denotes the corresponding spectral reflectance value for each band.

#### Indices-based feature extraction

For indices-based feature extraction, we utilise six different popular spectral indices, where 2 indices are related to the vegetation index, 2 are related to water, and the remaining 2 indices are related to soil and vegetation information (Eq. 3). It provides 6-D information on features in the form of indices-based feature set $F_3$.

3 $$\begin{aligned} F_3= \{\text {NDVI}, \text {RVI}, \text {SAVI}, \text {SATVI}, \text {NDWI}, \text {MNDWI}\}, \end{aligned}$$

where NDVI is the normalised difference vegetation index, RVI is the ratio vegetation index, SAVI is the soil-adjusted vegetation index, SATVI is the soil-adjusted total vegetation index, NDWI is the normalized difference water index, and MNDWI is the modification of normalized difference water index.

#### Schemes

We prepare seven different schemes to implement for LULC classification in our work. These seven different schemes include individual features such as spectral features, spatial features, and statistical features and their combinations. Detailed schemes are presented in Table 2.

#### Classification

We classify different LULCs of the City of Melbourne, Victoria using our proposed features and schemes. For the classification purpose, we utilise six popular ML algorithms: RF^48^, SVM with Linear kernel^48^, SVM with RBF kernel^48^, ANN^48^, NB^49^, and GLM algorithm^50^. We choose optimal hyper-parameters using a grid search technique for the ML algorithms. Here, the initial range of values is chosen based on the standard practice and empirical study. For the RF algorithm, we select a different number of trees (10, 20, 30, 40, 50, 60, 70, 80, 90, 100) and used the optimal trees as 100. Similarly, for the SVM with Linear kernel, we tune only C values in the range of 0 to 40 automatically. For SVM with RBF kernel, we obtain the automatically tuned Gamma and C parameters in the first 10 sets of combinations. For the ANN algorithm, we set different numbers of nodes in the hidden layer (1, 3,5, 7, 10, 15, 20, 25, 30, 35 and 40), and choose the best in our experiment. For the NB algorithm, we automatically tune three different hyper-parameters: kernel (true, false), fL (0, 1, 2, 3, 4, and 5), and adjusted (0, 1, 2, 3, 4, and 5). We do not need to tune the hyper-parameters for the GLM algorithm.

**Figure 3:**
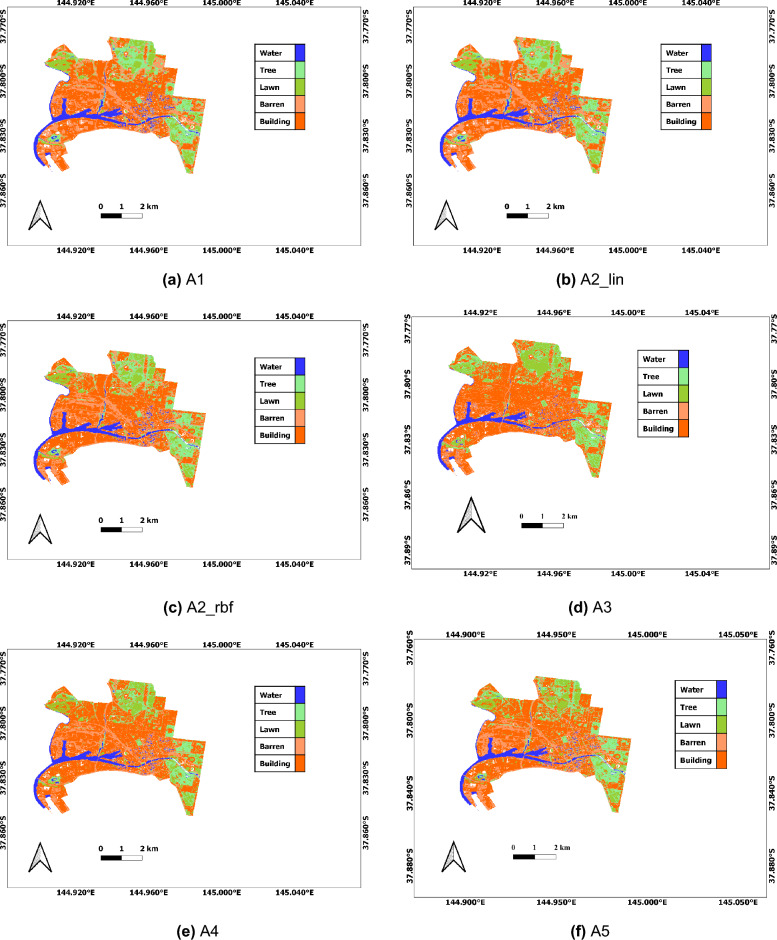
LULC maps of the study area using six different machine learning algorithms: (**a**) RF (A1), (**b**) SVM Linear (A2_lin), (**c**) SVM RBF (A2_rbf), (**d**) ANN (A3), (**e**) NB (A4), and (**f**) GLM (A5). Note that we use QGIS software^34^ (https://qgis.org/en/site/, version: 3.24.2)) for plotting.

**Table 4:** Average category-wise performance analysis using Precision (P), Recall (R), F1-score (F) and Accuracy (Acc.) of the best-performing ML algorithm (RF) with *Sp* + *St* + *Ind* Scheme.

Category	P	R	F	Acc.
Building	0.99	0.99	0.99	0.99
Barren	0.99	0.99	0.99	0.99
Lawn	1.00	1.00	1.00	1.00
Trees	1.00	1.00	1.00	1.0s0
Water	1.00	1.00	1.00	1.00

**Figure 4:**
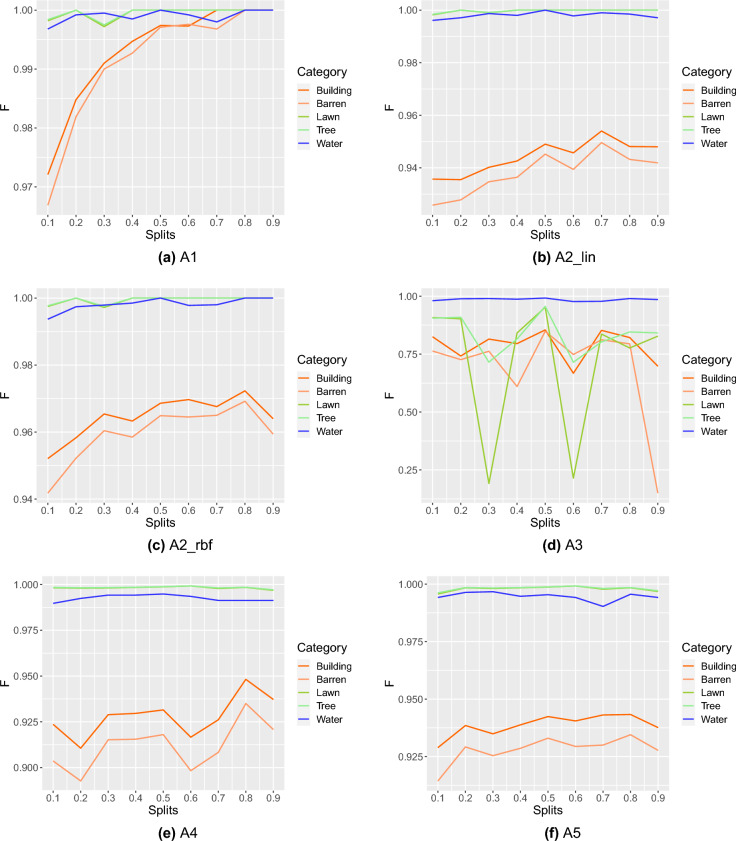
Robustness study at category-level of six different ML algorithms using F1-score (F): (**a**) A1, (**b**) A2_lin, (**c**) A2_rbf, (**d**) A3, (**e**) A4, and (**f**) A5.

## Results and discussion

### Comparative study of ML algorithms

We compare six different popular ML algorithms (RF, SVM Linear, SVM RBF, ANN, NB, and GLM) using all seven different schemes (Sp, St, Ind, Sp + St, Sp + Ind, St + Ind, and Sp + St + Ind) considered in our work. The detailed results are presented in Table 3. We notice that the 7th combination (Sp + St + Ind) scheme imparts stable performance for all six algorithms with an average of 0.95 accuracy. Richer information is extracted by the use of a combination of information than independent use of individual information. Similarly, we notice that the RF algorithm performs the best, yielding 0.99 accuracy, among others. The classified output map of the current study area is presented in Fig. 3. We speculate that ensemble learning algorithms such as RF have the ability to incorporate learned information hierarchically, thereby leading to overall performance improvements.

### Category-wise performance analysis

We analyse the category-wise performance achieved from the best-performing method (RF) for the LULC classification. We notice that the RF shows excellent averged performance for three categories—Lawn, Trees, and Water (Table 4). In contrast, it is slightly lower discriminant for the other two categories–Building and Barren. We suspect that this result is attributed to the similar reflectance patterns of Building and Barren regions. We also study individual fold’s performance using the confusion matrices, which are presented in the supplementary file (Pages 1–3, Lines 13–17, and Figs. 1 and 2). This also shows similar results.

**Figure 5:**
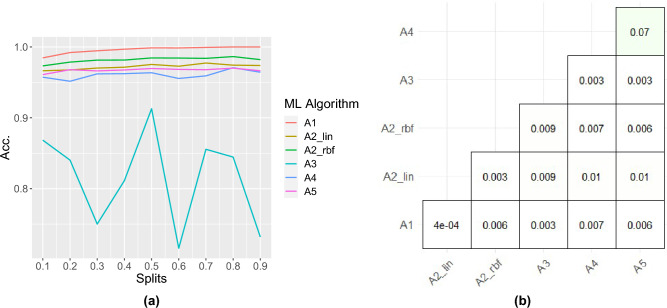
Robustness study of ML algorithms using overall classification accuracy (**a**) achieved from varying training splits against pair-wise statistical analysis (**b**) achieved from varying training splits.

Besides, we conduct two more studies at the category level: (1) variable importance study, and (2) model interpretability for generalisability. To understand the contribution of the variables, we calculate the variable importance using a fitted model with a 0 to 100 scale, where 0 is the lowest and 100 is the highest importance. The results are presented in the supplementary file (Pages 3–4, Lines 19–27, and Fig. 3). The result shows that the different categories prefer different variable combinations, thus suggesting to use all combinations to maximise the performance. Furthermore, we utilise the averaged SHapley Additive exPlanations (SHAP) interpretation technique for generalisability. The results are presented in the supplementary file (Pages 5–8, Lines 29–39, Figs. 4, 5, 6, 7, and 8). From this result, we find that different variables are contributing to different categories for performance improvement as in the variable importance study. This finding helps generalise our proposed approach for the LULC classification problem after the identification of the best variables and models.

### Robustness study at category-level

We study the robustness of ML algorithms using varying amounts of training data at the category level. Based on the performance trend using F1-score (F) against varying training data, we find the robustness of the ML algorithms for each category. The results are presented in Fig. 4, where we observe that the RF (A1) is the best-performing algorithm over five different categories in terms of stable performance against increasing training splits. This result also highlights that three categories (Water, Lawn, and Tree) are highly separable irrespective of the varying amount of training data. We also notice that the performance of all five categories converges at a single point after 0.8 training data split. Further, ANN (A3), which falls into the categories of DL algorithms, is the worst-performing algorithm. Here, we observe that it imparts highly fluctuating performance over four categories (Building, Barren, Lawn, and Tree) irrespective of increasing training splits except for the Water category. We believe that this result is attributed to the imbalanced dataset.

### Robustness study at overall classification level

The robustness of the ML algorithms at the overall classification level is crucial in terms of overall performance because category-level robustness might not always necessarily suffice for the overall classification-level robustness. This can be achieved using overall classification performance trend analysis against varying amounts of training datasets as in Fig. 5. From the figure, we observe that the RF (A1) is the best-performing algorithm with its stable and consistent classification accuracy against varying training splits compared to other algorithms. Whereas, the ANN (A3) is the least-performing algorithm with its highly fluctuating/unstable classification accuracy despite increasing training data size.

### Statistical analysis of the performance

To further analyse the significance of the performance measures produced by ML algorithms, we perform the statistical analysis of accuracy measures. For this, we follow two steps. First, we test the normality using JB-test which identifies that our data distribution is non-parametric. Second, we perform the non-parametric statistical test using Wilcoxon ranked test between ML algorithm pairs. The results are presented in Fig. 5, where we find that the classification performance of our proposed feature extraction method is significant (*p* value $\le$ 0.05) with ML algorithms (RF, SVM Linear, SVM RBF, ANN) except with A4 (NB) and A5 (GLM).

## Conclusion and future works

Our study concludes that utilising a combination of spatial, statistical, and indices-based schemes results in a stable/reliable performance with ML algorithms except for ANN, at both category-level and overall LULC classification levels, focusing on smart city planning. Furthermore, we observe that traditional ML algorithms provide greater robustness compared to the DL-based method (ANN) when dealing with imbalanced and/or limited datasets. Further, our findings specify that the suggested schemes, which are explainable and interpretable, positively impact the performance of ML algorithms, as shown through the statistical significance tests. Nevertheless, it is important to note that our approach’s effectiveness may vary in other geographic locations/areas considering the geographic variability. Therefore, in benchmarking the method devised in this study, further implementation in varieties of locations is recommended.

### Supplementary Information

Supplementary Information.

## Data Availability

We collected the dataset for our study using the Copernicus Open Access Hub repository.
