# Supplementary material for: Land use and land cover (LULC) performance modeling using machine learning algorithms: a case study of the city of Melbourne, Australia
Source: Sci Rep. 2023 Aug 19;13:13510. doi: 10.1038/s41598-023-40564-0 (PMC10439905; doi:10.1038/s41598-023-40564-0)
Supplement: Supplementary file 1 — Supplementary Information. [file 41598_2023_40564_MOESM1_ESM.pdf]

Supplementary file: Land Use and Land Cover (LULC) Performance Modeling Using Machine Learning Algorithms: A Case Study of City of Melbourne, Australia

Jagannath Aryal[1,*,+], **Chiranjibi Sitaula**[1,+], **and Alejandro C. Frery**[2]

[1]Earth Observation and AI Research Group, The University of Melbourne, Infrastructure Engineering, Melbourne, 3800, Australia
[2]Victoria University of Wellington, School of Mathematics and Statistics, Wellington, 6012, New Zealand
[*]Corresponding: jagannath.aryal@unimelb.edu.au
[+]these authors contributed equally to this work

ABSTRACT

This document contains supplementary information related to the main paper. In particular, it comprises a category-wise analysis using a confusion matrix, variable importance study and explainability with the SHapley Additive exPlanations (SHAP) with the LULC classification in the context of the City of Melbourne, VIC, Australia.

Confusion matrix

We employ the confusion matrix for the best-performing model (RF) to have a better understanding of the category-wise distribution of the performance during classification on 10 different folds (Figs. 1 and 2). From the figures, we observe that the proposed approach delivers strong discriminability (e.g., Fold 1 (a) provides overall accuracy: 99.89%, producer's accuracy (Barren): 99.68%, and user's accuracy (Barren): 99.83%) across all five categories during classification, improving the performance subsequently.

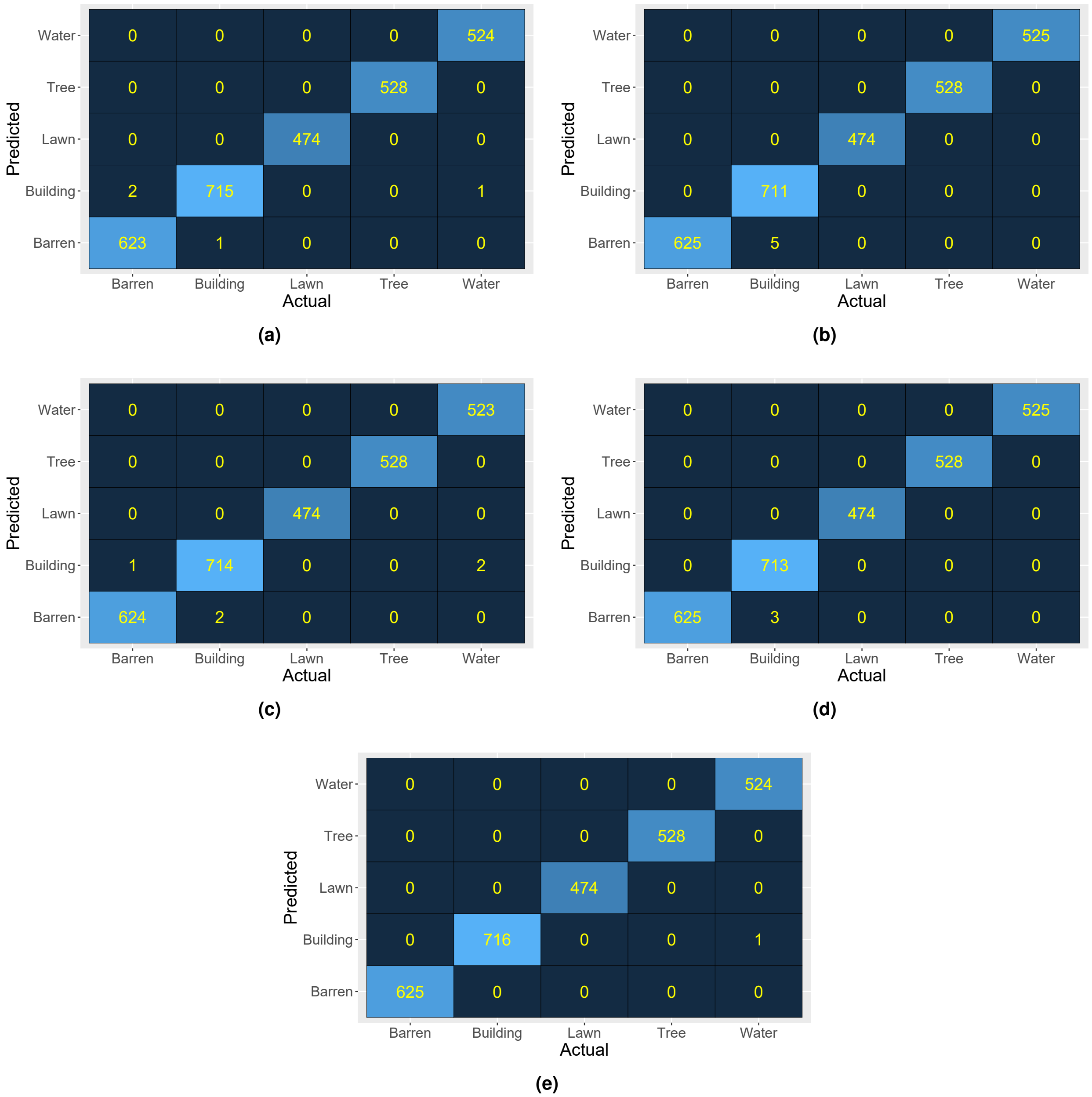

Figure 1. Confusion matrix produced by RF: (a) Fold 1, (b) Fold 2, (c) Fold 3, (d) Fold 4, and (e) Fold 5.

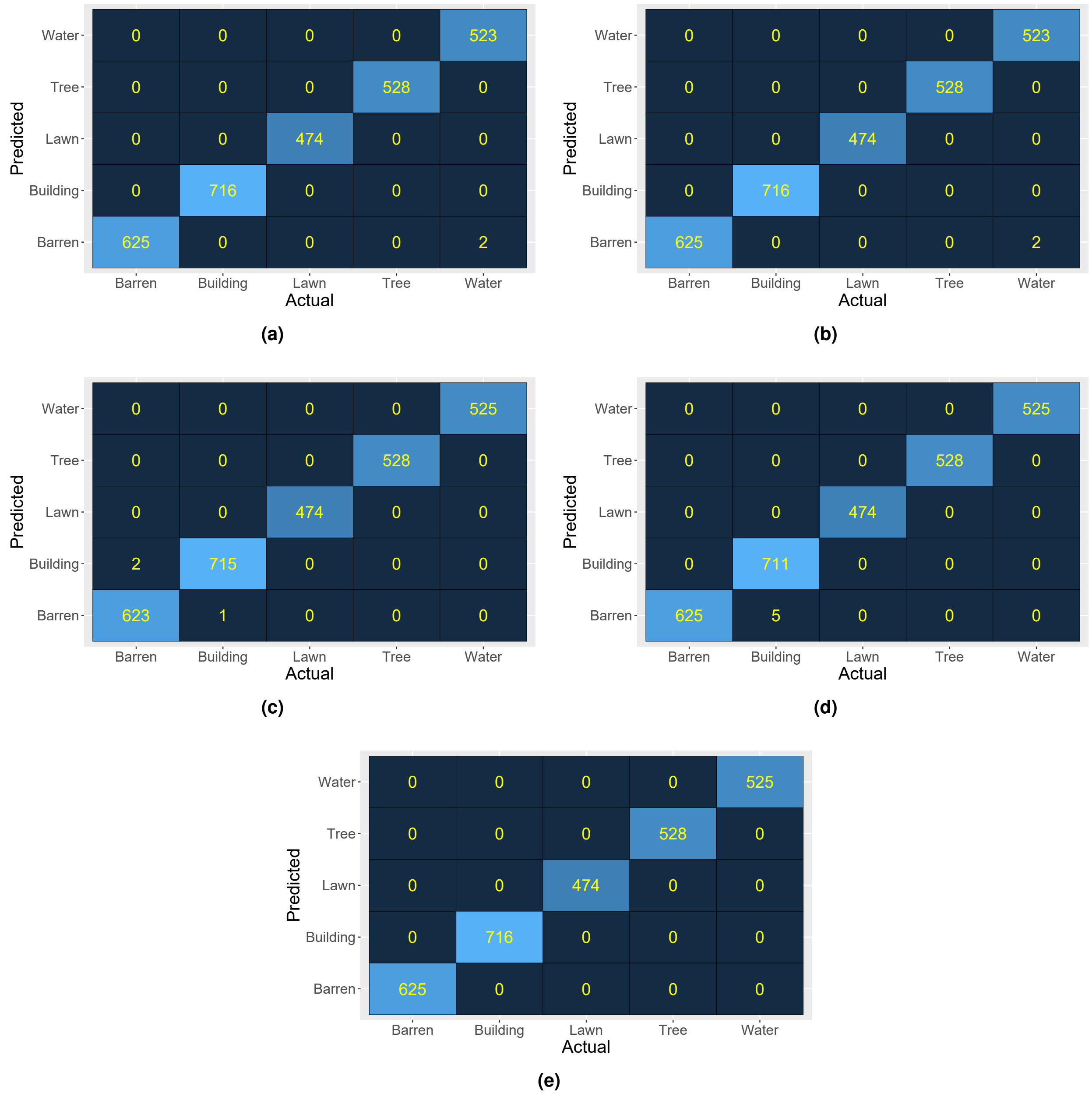

Figure 2. Confusion matrix produced by RF: (a) Fold 6 (b) Fold 7, (c) Fold 8, (d) Fold 9, and (e) Fold 10.

Variable importance

The variables involved in the combination of spectral, spatial and indices-based information are responsible for both overall and category-wise classification performance improvement. Therefore, to understand which variable combinations are important for which category, we perform the variable importance analysis with the fitted model (RF) using caret package[1] in R, where the variable importance score ranges from 0 (the lowest) to 100 (the highest). The result is presented in Fig. 3. From Fig. 3, We find that the importance/performance of variable combinations differs from one category to another. For instance, in the 'Barren' category, the combination of variables such as NDWI (indices-based), X56.1 (spectral), and layer.2 (statistical) are more crucial. Whereas, for the 'Lawn' category, the variables such as X56.10 (spectral), layer.4 (statistical) and NDVI (indices-based) are more prominent. Therefore, given the importance/performance of such varying variable combinations for each category, we utilise all combinations to maximise classification performance.

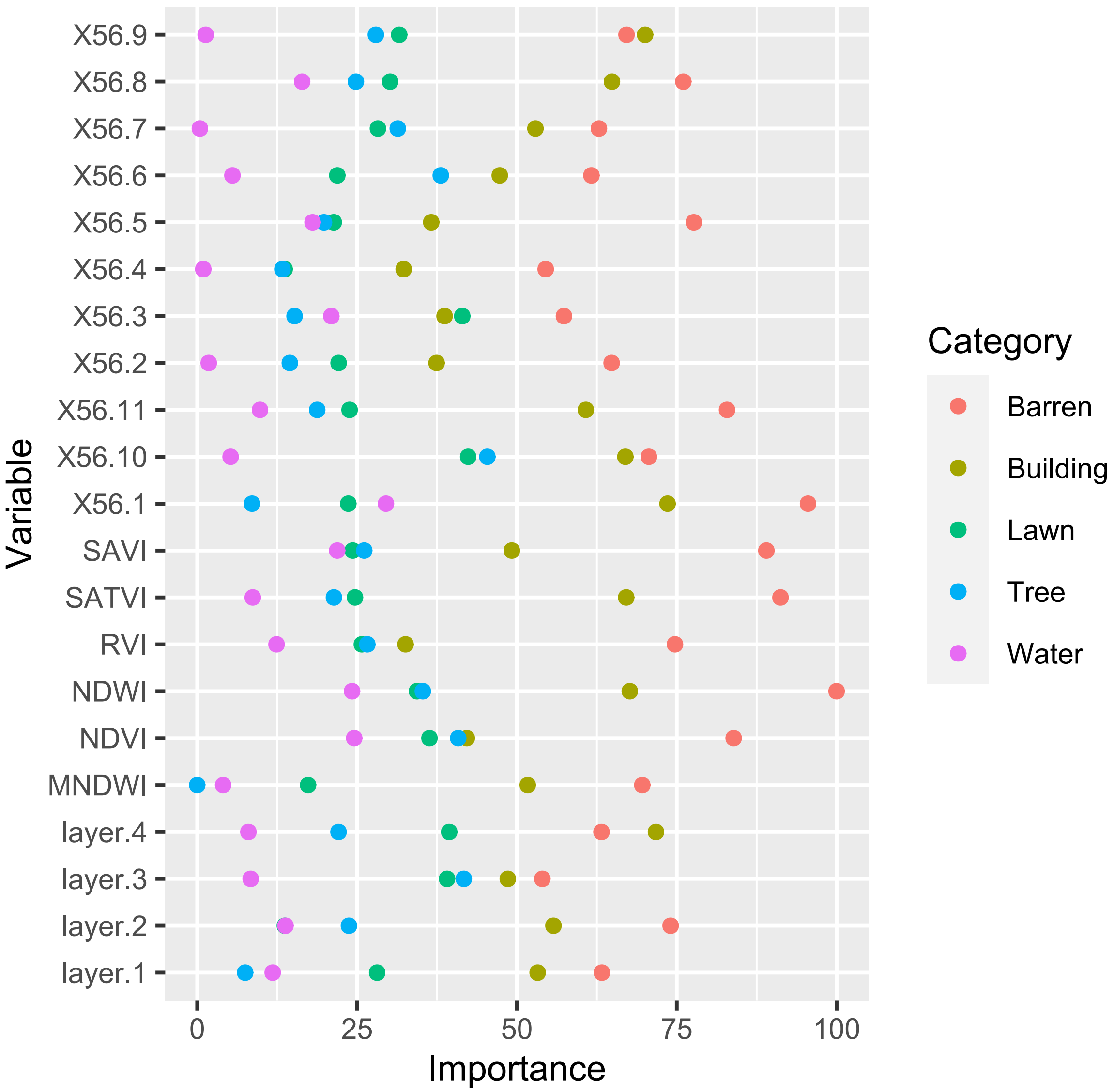

Figure 3. Category-wise variable importance (higher the most important) based on the RF algorithm. Note that the variables starting from 'X' are spectral-based, whereas the variable names starting from the 'layer' are statistical-based and the remaining variables are indices-based.

Model Interpretability

To show the contribution of each variable at a further finer level utilised in this study, we extract the averaged SHapley Additive exPlanations (SHAP) or also called shapely value[2] for them using the 'fastshap' package in R[3]. The SHAP is used for the local interpretability of the machine learning algorithms. Higher SHAP gives higher importance to discriminating among categories. Therefore, we perform the category-wise averaged SHAP calculation (Figs 4, 5, 6, 7, and 8 for Barren, Building, Lawn, Tree, and Water, respectively) on one of the test folds used in our study. From the figures, we find that the importance of the variables is dependent on the category. For example, for the 'Barren' category, the 'NDWI' variable shows a higher impact (higher SHAP value) for the classification, whereas it is not the case for other categories. This shows that the highly-discriminating variables are different across five different categories. Further, we extract the SHAP values of the highly-discriminating variable across the testing samples falling into the corresponding category. This result unveils that the highly discriminating variable imparts higher SHAP values under the corresponding category. Based on such findings, we could generalise our proposed model for the LULC classification problems.

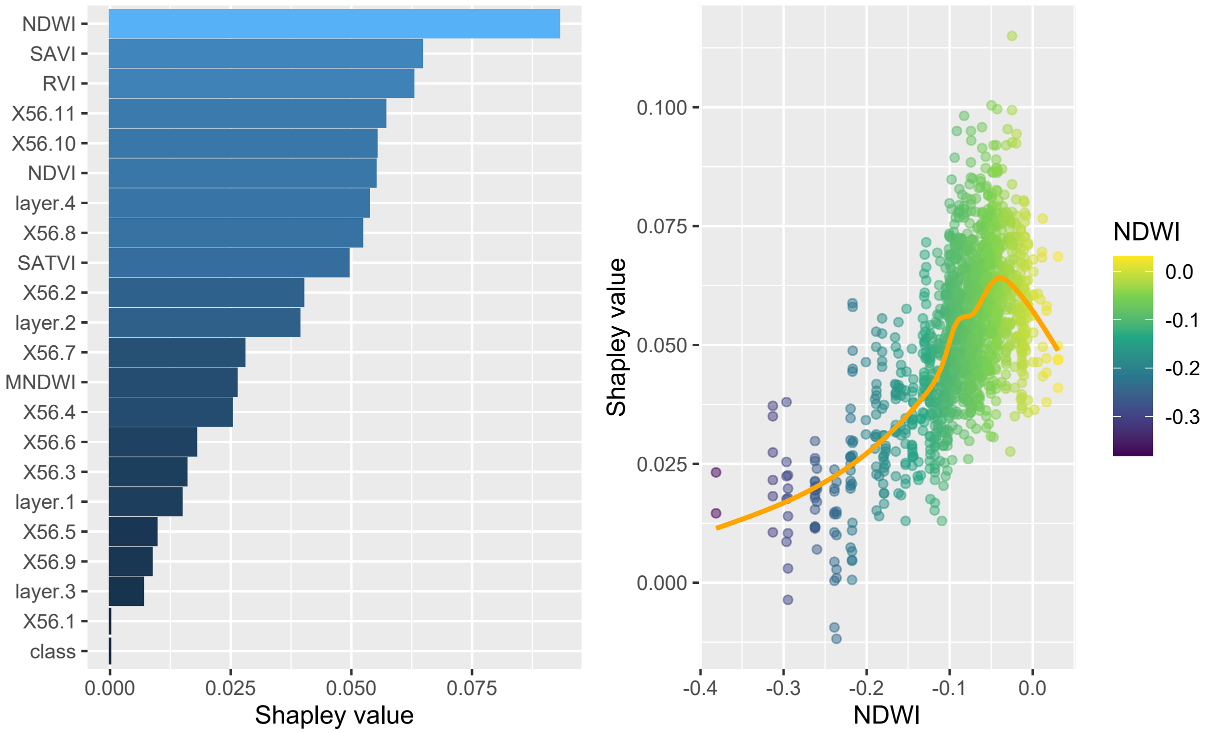

Figure 4. Averaged shapely values of the corresponding variable belonging to the 'Barren' category from the left plot. Also, the right-hand side plots the individual SHAP values of the NDWI (highest SHAP value) for the samples belonging to the corresponding category on the testing fold.

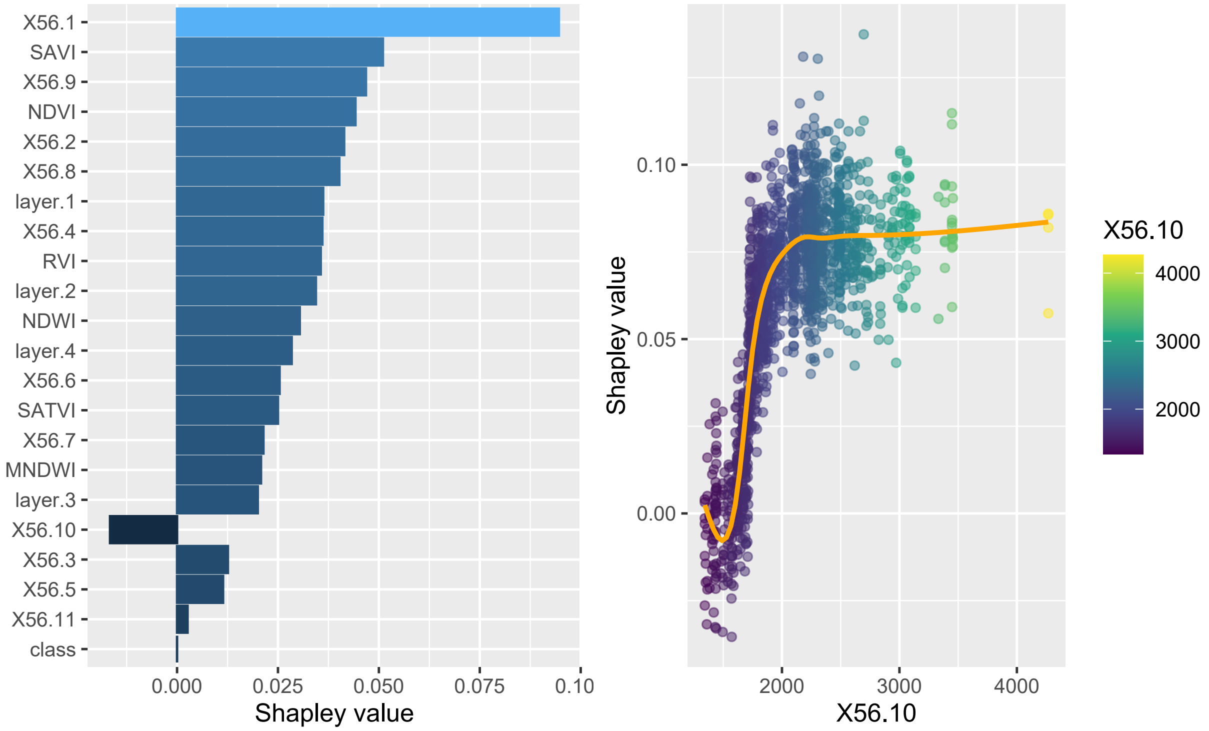

Figure 5. Averaged shapely values of the corresponding variable belonging to the 'Building' category from the left plot. Also, the right-hand side plots the individual SHAP values of the X56.10 (highest SHAP value) for the samples belonging to the corresponding category on the testing fold.

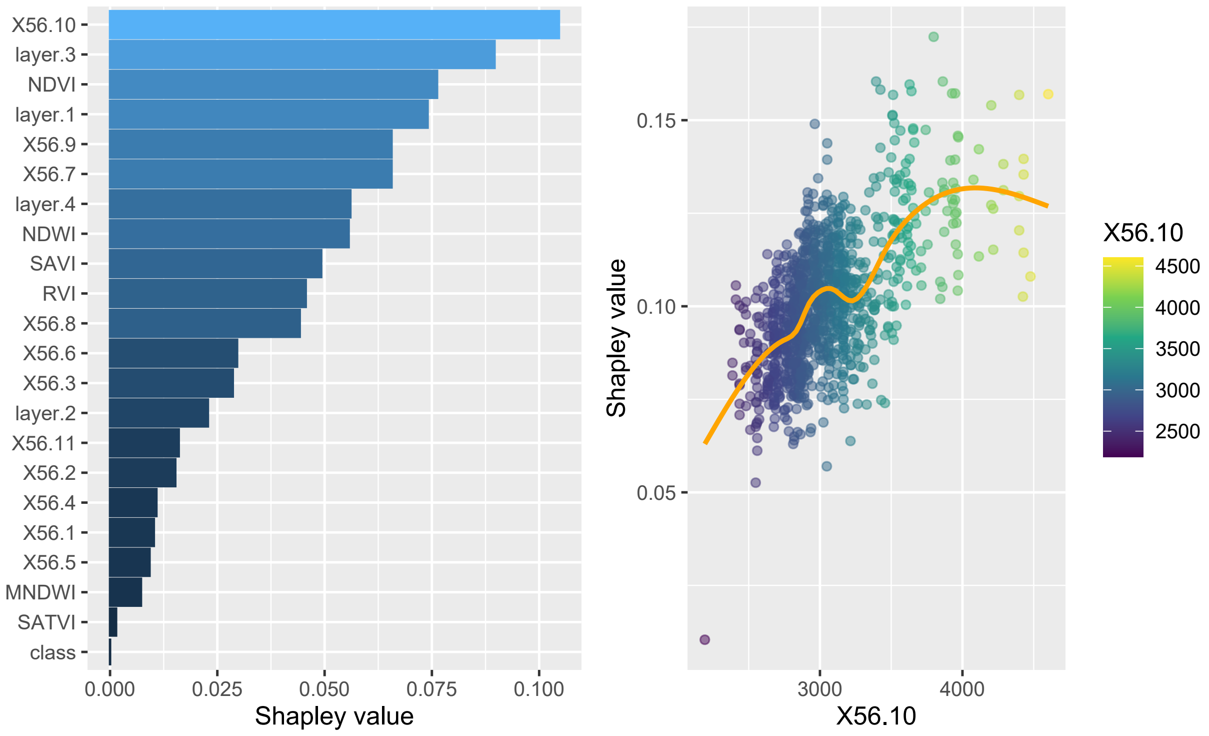

Figure 6. Averaged shapely values of the corresponding variable belonging to the 'Lawn' category from the left plot. Also, the right-hand side plots the individual SHAP values of the X56.10 (highest SHAP value) for the samples belonging to the corresponding category on the testing fold.

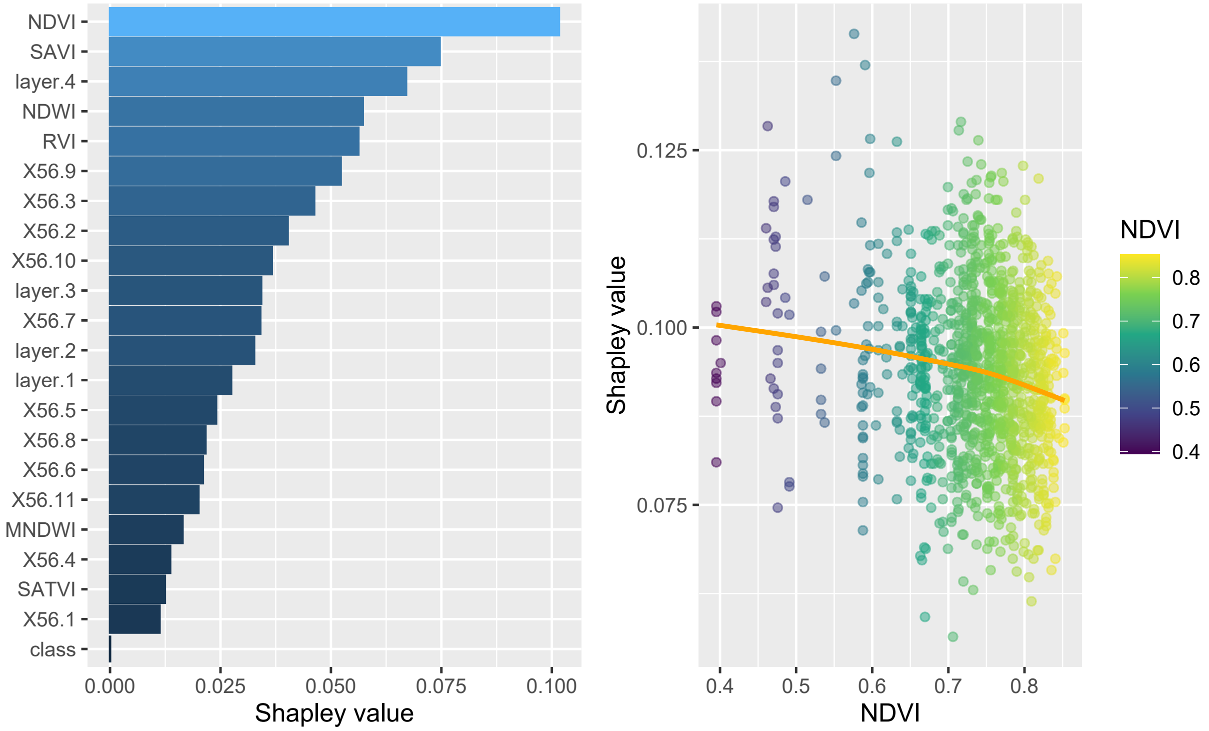

Figure 7. Averaged SHAP values of the corresponding variable belonging to the 'Tree' category from the left plot. Also, the plot on the right-hand side plots the individual SHAP values of the NDVI (highest SHAP value) for the samples belonging to the corresponding category on the testing fold.

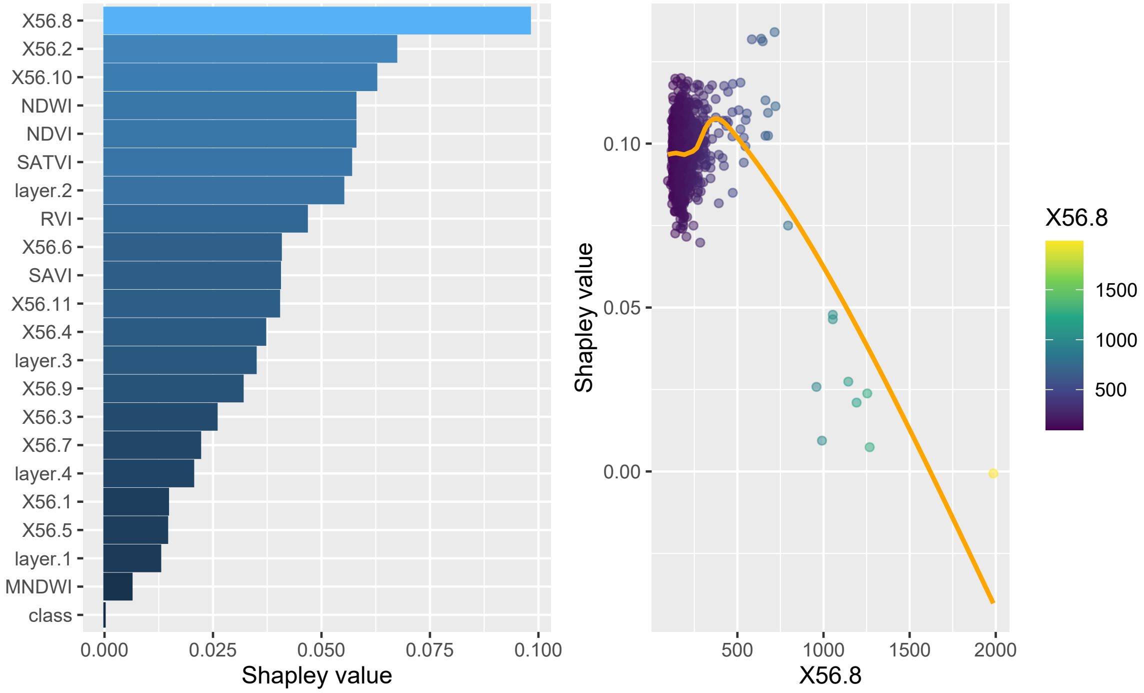

Figure 8. Averaged SHAP values of the corresponding variable belonging to the 'Water' category from the left plot. Also, the right-hand side plots the individual SHAP values of the X56.8 (highest shapely value) for the samples belonging to the corresponding category on the testing fold.

References

1. Kuhn & Max. Building predictive models in r using the caret package. *J. Stat. Softw.* **28**, 1–26, DOI: 10.18637/jss.v028.i05 (2008).
2. Lundberg, S. M. & Lee, S.-I. A unified approach to interpreting model predictions. *Adv. neural information processing systems* **30** (2017).
3. Greenwell, B. *fastshap: Fast Approximate Shapley Values* (2020). R package version 0.0.5.
